# Frontiers in Parasitology Grand Challenge

**DOI:** 10.3389/fpara.2022.902098

**Published:** 2022-04-28

**Authors:** Alex Loukas

**Affiliations:** Centre for Molecular Therapeutics, Australian Institute of Tropical Health & Medicine, James Cook University, Cairns, QLD, Australia

**Keywords:** parasites, parasitology, immunity, protozoans, helminths, vaccines, experimental infection, climate change

## Introduction

Parasites are the most prevalent group of eukaryotic organisms on the planet. Humans harbor more than 300 species of helminths and 70 species of protozoans (Cox, 2002). Some of these parasites are rare or accidental passengers, but at least 90 parasites are relatively common inhabitants of the human body. If the other 65,000 species of known vertebrates house a similar number of different parasites, then we are talking about almost 6 million different types of parasites in vertebrate hosts alone. Admittedly, some parasites infect multiple different hosts, but nonetheless, their diversity is impressive. Then consider the 1.3 million known invertebrate animals, most of which also harbor multicellular and unicellular parasites, and then the more than 300,000 plants and their parasites, and the concept becomes slightly overwhelming.

Sadly, despite the prevalence and importance of parasites to human and animal health, there are very few examples of commercially available vaccines against parasitic diseases. In this COVID pandemic era as we witnessed anti-viral vaccines go from bench to bedside in a matter of months, it is all the more remarkable that we have so few anti-parasite vaccines despite knowing of their importance for thousands of years.

## History

The first human parasites to be described were helminths because of their large size and visibility to the naked eye. In the Ebers Papyrus of 1500 BC, Egyptian scholars noted parasitic helminths, and even recommended treatment with Aloe vera. Indeed, schistosome eggs have been identified in 5,000-year-old Egyptian mummies (Barakat, 2013). The Ancient Greeks also wrote profusely on parasites, again with the focus on macroscopic worms in this pre-microscopy era. Aristotle was fascinated by the helminths, and held strong but often factually incorrect opinions. In his *Historia animalium*, Aristotle accurately verified earlier observations of Hippocrates on the existence of “the flatworm,…the roundworm… and the ascarid” but little did he know how far off track he was when he wrote “These intestinal worms do not in any case propagate their kind” (Stewart, 1951). More than 500 years later the Greeks still had not come to terms with the reproductive processes of helminths. Galen (130–200 AD) described the origin of the “broadworm”—likely what we now know as the tapeworm—as “…the changing of a small skin within the intestines into a certain living body, that causes continual gnawings at the stomach….” We now know that these fascinating, even beguiling parasites are not derived from our own tissues, and are indeed highly fecund, shedding tens-to-hundreds of thousands of eggs per day.

## Where Are All The Vaccines?

The sheer diversity of parasitic organisms, the inhospitable niches they have evolved to occupy, and the remarkable strategies they have developed to hijack various host physiological processes are purely breath-taking. While it is easy to be captivated by the wonderful world of parasites and the various evolutionary events that have independently led to parasitism over and over again, it is sobering to consider their enormous health impact. Despite billions of dollars of investment, the only malaria vaccine on the market confers modest efficacy at best (Zavala, 2022), and there are no human vaccines for diseases caused by the other major unicellular parasites, such as trypanosomes and *Leishmania*. Indeed, the most promising approach to vaccination against leishmaniasis is a novel strategy that induces humoral immunity against the sandfly vector salivary proteins to prevent establishment of an immune privileged environment at the bite site (Aoki et al., 2022). To think that a single celled parasite would take up residence in the very cell designed to recognize and help kill it (antigen presenting cells) just highlights the myriad sophisticated means by which parasites evade our best attempts to remove them. It is no wonder that anti-parasite vaccines are lagging behind those against viral and bacterial diseases.

The situation is no better for vaccines against multicellular parasites. There are no anti-helminth vaccines on the market for humans, although a small number of veterinary vaccines have been developed, including highly efficacious subunit vaccines against porcine tapeworm infection (Lightowlers et al., 2021). It should be noted however that most of the anti-helminth livestock vaccines that are available are designed to accelerate naturally acquired immunity that normally develops after initial exposure early in life when animals are most susceptible and productivity losses are highest. Protective immunity is slow to develop for most of the human helminths, and is not sterilizing in nature, but rather enables a status quo whereby chronic infection establishes at intensities that are tolerated by most individuals (Loukas et al., 2021). Efforts are underway to develop subunit vaccines for the major human helminths (Zawawi and Else, 2020; Zhang et al., 2020), but progress is slow, and some of the most promising approaches are based on old technologies (Chapman et al., 2021b) that are difficult to scale-up in a cost-effective manner.

So, why are there so few anti-parasite vaccines? From a personal perspective, it comes down to two major reasons: (i) insufficient funding due to the nature of the market (i.e., those in most need are least likely to afford the solution), and therefore too few active researchers; (ii) the developmental and molecular sophistication of these hitchhikers has ensured that our best immunological attempts to eliminate them have been, mostly, futile.

## Grand Challenge 1: Anti-Parasite Vaccines

Are vaccines really the panacea of control for parasitic diseases, or is money and effort better spent on other control and elimination approaches? If we can get COVID vaccines on the market so rapidly, why can't we do the same for parasitic diseases? Is it just a matter of more money, and therefore more R&D? Do new tools in vaccinology, such as mRNA vaccine technology have a place in parasitology, and will it make a difference?

### The Good Side of Parasitic Infections

Parasites are so intricately interwoven into our very fabric, and millennia of host-parasite coevolution has frequently hit upon unique molecular approaches to allow parasites to evade immunity and other hostilities that our bodies throw at them. The very property of parasites that makes vaccine development so challenging—their immunoevasive capacity—can be exploited to gain unique insights into which pathways to target for controlling chronic non-infectious diseases, particularly those underpinned by inflammation and metabolic disturbances. Parasitic infections, or more accurately, the immune responses they invoke, have been shown to protect against a diverse range of infectious and non-infectious diseases. In 1917, Julius Wagner-Jauregg induced fever in neurosyphilis patients by infecting them with *Plasmodium* malaria parasites (Karamanou et al., 2013). He received the Nobel Prize for this work because neurosyphilis was dreadful and incurable, and “desperate maladies justify desperate remedies” was the prevalent notion at the time (Raju, 1998). More recently, the potential of helminth therapy for treating non-infectious inflammatory diseases has been recognized (Maizels, 2016), and indeed the ability of some helminths to suppress severe COVID by dampening pulmonary inflammation has been reported (Wolday et al., 2021). These findings are drawing fresh attention to parasitology through a different lens, and the notion of parasite therapy warrants much greater consideration than it currently receives. Some unicellular parasites are highly genetically tractable (Briquet et al., 2022), and recent advances in helminth transgenesis (Ittiprasert et al., 2019) is showing promise.

## Grand Challenge 2: Parasite Derived Therapies for Inflammatory Disorders

Can helminths or helminth-derived/inspired be developed as next-generation therapeutics or prophylactics to treat diseases that result from a dysregulated immune system, whether they by non-communicable such as autoimmunity and allergy, or the complications of communicable diseases such as severe COVID? To extend the challenge, could parasites that have been shown to be safe and well-tolerated in experimental human infection studies (see next paragraph) be engineered to secrete therapeutic or prophylactic molecules to further enhance their endogenous medicinal properties?

### Human Experimental Infection Studies

Both of the Grand Challenges outlined above can be addressed more rapidly now than they could 20 years ago with advances in human challenge models for some of the major parasitic diseases. Vaccines and drugs can be tested much more rapidly in a controlled environment with experimental infections of otherwise healthy volunteers than by testing them in disease endemic countries via natural field exposure (Roestenberg et al., 2018a,b; Cooper et al., 2019; Langenberg et al., 2020; Chapman et al., 2021a,b). Controlled human infections with *Plasmodium falciparum, Necator americanus* and *Schistosoma mansoni* have been shown to be safe and well-tolerated when intervention is carefully controlled, and have been approved by the major regulatory agencies around the world for clinical trial use. Nonetheless, there remain major ethical considerations (Elliott et al., 2018). There is real potential for parasites to be used prophylactically for a range of disorders, including the metabolic syndrome pandemic that has emerged over the past decade (Rennie et al., 2020). To borrow a phrase from my friend and colleague, John Croese, perhaps “parabiotics” have a place in resetting our metabolic and immune systems? The human microbiota has received so much attention of late, so why shouldn't the “macrobiota” be treated with equal reverence? Indeed, many authors have shown that the macrobiota strongly influences (even orchestrates) the composition of the microbiota (Giacomin et al., 2015; Rapin and Harris, 2018; Moyat et al., 2019), further highlighting the notion that humans and animals are merely vessels for the smaller organisms that live inside and control them.

## Grand Challenge 3: Parasites and Climate Change

The third and final Grand Challenge in Parasitology that I would like to address is that of climate change. As the planet warms, parasites are spreading to areas that were previously inhospitable for them (Kubelka et al., 2022). Host and vector distributions are shifting, and changing environmental conditions are allowing some parasites with external developmental stages to extend their distribution into areas that were previously inhospitable (usually too cold) (Short et al., 2017). For example, gastrointestinal nematodes of livestock are moving into areas in Europe that were previously thought to be too cold (Rose et al., 2016). Moreover, heat stress in areas that are becoming increasingly warmer negatively impacts the ability to hosts to control parasitic infections (Bautista-Garfias et al., 2022). The dependence of humans on their livestock and crops, particularly for subsistence farmers in developing countries with few dietary options, means that a warming climate has devastating consequences for economic growth in these already impoverished regions. As efforts to scale-up and diversify aquaculture production gain momentum, rising sea temperature is negatively impacting fish farming, exemplified by increased susceptibility to parasitic diseases (Cascarano et al., 2021; da Costa et al., 2021).

### Frontiers in Parasitology Is Open for Business

Frontiers in Parasitology and its dedicated specialty sections will become a forum for all manner of research, review and commentary articles to address these and other perceived Grand Challenges in parasitology. The journal is by no means limiting itself to the topics I chose to address here—these are just ones that are close to my heart. There is an exhaustive list of important subject matters in the various disciplines of parasitology, and Frontiers in Parasitology is squarely situated to be the venue for the most cutting-edge advances and associated discourse in all areas of the field.

## Author Contributions

The author confirms being the sole contributor of this work and has approved it for publication.

## Conflict of Interest

The author declares that the research was conducted in the absence of any commercial or financial relationships that could be construed as a potential conflict of interest.

## Publisher's Note

All claims expressed in this article are solely those of the authors and do not necessarily represent those of their affiliated organizations, or those of the publisher, the editors and the reviewers. Any product that may be evaluated in this article, or claim that may be made by its manufacturer, is not guaranteed or endorsed by the publisher.
